# Understanding the causes of high mortality among adult acute leukaemia patients in Armenia

**DOI:** 10.3332/ecancer.2025.1952

**Published:** 2025-07-23

**Authors:** Astghik Voskanyan, Lusine Harutyunyan, Arusyak Ivanyan, Alisa Movsisyan, Nerses Ghahramanyan, Lusine Sahakyan, Shushan Hovsepyan, Samvel Danielyan, Hayk Grigoryan, Gevorg Tamamyan

**Affiliations:** 1 Immune Oncology Research Institute, Yerevan 0014, Armenia; 2 Yeolyan Hematology and Oncology Center, Yerevan 0014, Armenia; 3 Pediatric Cancer and Blood Disorders Center of Armenia, Yeolyan Hematology and Oncology Center, Yerevan 0014, Armenia; 4 Yerevan State Medical University, Yerevan 0025, Armenia

**Keywords:** leukaemia, hematology, mortality, low-middle income country, resource-limited setting

## Abstract

**Background:**

Acute leukaemias (AL) are a group of heterogeneous malignancies characterised by clonal hematopoietic progenitor cell proliferation. Despite advances in the therapy of acute lymphoblastic leukaemia (ALL) and acute myeloid leukaemia (AML) in developed countries, outcomes in developing countries like Armenia are thought to be substantially worse.

**Aims:**

The aim of this article is to understand how the limitations in diagnostic and treatment modalities in Armenia impact the clinical outcomes of AL patients and by presenting it to health authorities contribute to the development of evidence-based policies and regulatory improvements to enhance patient care.

**Methods:**

We interrogated data from 431 adults representing all cases of adult AL in Armenia at the Hematology Centre of Armenia from 1 January 2016, to 31 December 2020. Death data were obtained from the United Information System of Electronic Healthcare in the Republic of Armenia. There were some limitations with data collection due to the absence of a unified electronic database and detailed paper records.

**Results:**

Over the 5 years a total of 431 patients were diagnosed with AL at the Hematology Centre of Armenia of which 310 (72%) died. Male patients’ number was 131 (54%). Median age was 59 years (Interquartile Range = 50, 18–85 years). A morphological complete remission before death was reached in 82 (34%) patients, including 24 dying without relapse. Additionally, 50 subjects (20%) died during induction chemotherapy including 9 with ALL, 37 with AML and 1 with mixed-lineage leukaemia. Causes of death included no response to treatment (*N* = 29) or therapy-related complications including septic shock (*N* = 5), acute heart failure (*N* = 5), brain hemorrhage (*N* = 2) and acute respiratory failure (*N* = 1). Causes of death were unclear in eight patients. Thirty subjects failed induction therapy and declined further treatment before starting the induction. In 24 subjects’ remission, state and death causes were unclear. Before starting chemotherapy 58 subjects died, 26 of whom refused therapy and 24 had leukaemia progression. A 5-year survival was 22% including 26% for ALL and 21% for AML.

**Conclusion:**

The results of AL therapy in Armenia are worse than those reported in developed countries, where overall survival is about 60%. The major reasons are leukaemia progression and treatment-related complications.

## Introduction

Acute leukaemias (AL) are a group of heterogeneous malignancies characterised by clonal hematopoietic progenitor cell proliferation. The incidence rates of AL vary depending on various factors, including leukaemia subtype, gender, age and geographic location. There are two major categories, acute myeloid leukaemia (AML) and acute lymphoblastic leukaemia (ALL), each representing distinct epidemiological patterns. AML typically affects individuals with a median age of 66, with an incidence of 1.78 per 100,000 in those under 65. This rate escalates to 16.8 per 100,000 in those aged 65 and older, as reported in a 4-year dataset from the USA [1]. Conversely, ALL has a higher incidence in children, characterised by an age-adjusted rate of 1.5 per 100,000 in the USA [1].

Historically, in the absence of chemotherapy, individuals diagnosed with AL faced a dismal prognosis, with life expectancy limited to 6 weeks [2, 3]. However, the landscape of AL has undergone a remarkable transformation in recent years, thanks to the advent of chemotherapy and other therapeutic approaches, including allogeneic stem cell transplantation (allo-SCT). As a result, a significant proportion of patients now achieve complete remission (CR) and experience long-term survival, akin to achieving a potential cure [1, 2, 4]. Remarkably, the 5-year overall survival (OS) rate for paediatric ALL patients has reached an impressive 90%, while among adults, it ranges from 47% to 51% [4, 5]. For AML patients below the age of 45, the 5-year OS rate stands at 50%, but it diminishes significantly to a mere 2% for patients aged 75 and above [2]. These advancements underscore the evolving landscape of AL management.

Over the past few decades, the landscape of AL management has been transformed with the introduction of several Food and Drug Administration (FDA)-approved targeted therapies. These advancements have significantly improved the prognosis for patients with AL, particularly in cases of AML. Notable targeted therapies include FLT3 inhibitors (such as midostaurin, gilteritinib and quizartinib), isocitrate dehydrogenase (IDH) inhibitors (ivosidenib and enasidenib) and the use of the CD33-targeted monoclonal antibody gemtuzumab ozogamicin (GO) in specific cases [6]. In the context of ALL, several new therapies, including blinatumomab, inotuzumab ozogomycin and CAR-T therapy, have been introduced mainly as bridge therapies to allo-SCT [7–10]. However, it is important to recognise that AML and ALL continue to represent significant public health challenges globally [1], with more favourable outcomes typically observed in developed countries. Alarmingly, approximately 70% of cancer-related deaths occur in low- and middle-income countries [11], often due to limited access to modern treatment modalities. Developed regions allocate 5 to 10 times the per capita resources for cancer control compared to their counterparts in low- and middle-income countries. Importantly, the financial gap between developed and developing nations in this context is widening [11]. The primary objective of our study is to present a comprehensive assessment of the leukaemia landscape in Armenia while highlighting the substantial disparities that profoundly affect treatment outcomes. Through an analysis of the challenges faced in managing AL patients in Armenia, we aim to contribute to the development of holistic strategies aimed at ensuring equitable access to appropriate care for individuals, irrespective of their socioeconomic status.

## Materials and methods

This retrospective cohort study encompasses a cohort of 431 AL patients diagnosed at the Hematology Centre between 2016 and 2020, which draws on data from diverse sources, including outpatient and dispensary records, hospitalisation logs and clinical data obtained from the Registry of Blood Diseases of the Centre. The Hematology Centre stands as the only institution in Armenia dedicated to the treatment of leukaemia, thereby our dataset represents the entire nation. Death dates were taken from the United Information System of Electronic Healthcare in the Republic of Armenia. Survival analysis was performed through the Kaplan–Meier method.

## Results

**Figure 1.**
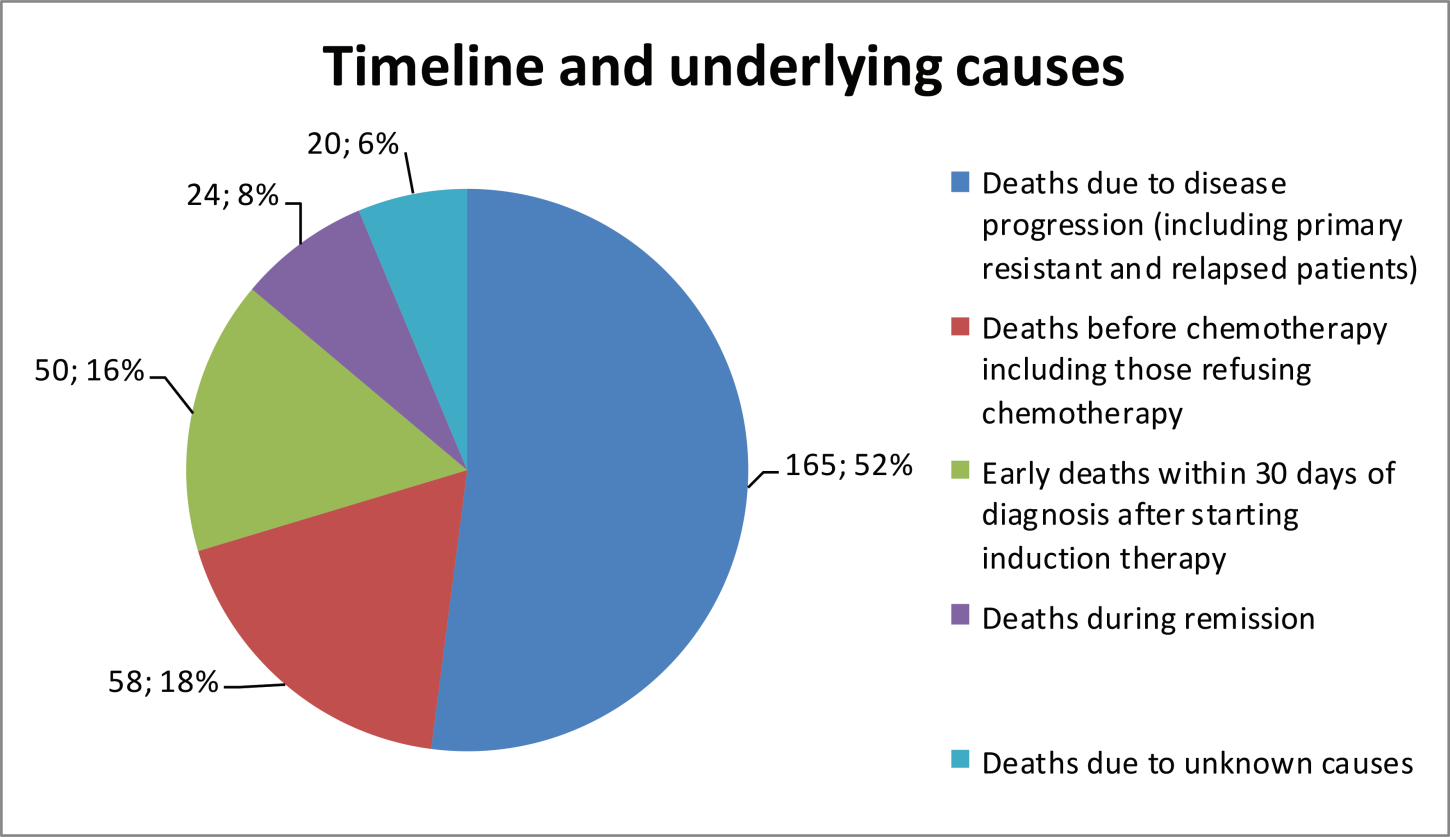
Mortality patterns and causes in AL patients.

**Figure 2.**
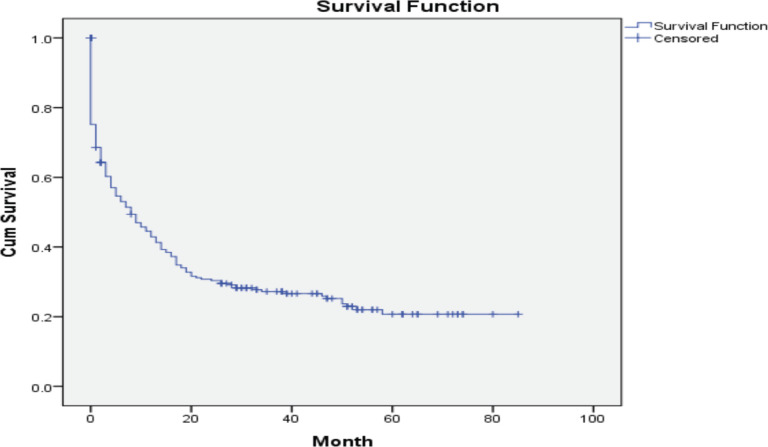
Five-year OS of patients with AL in Armenia.

Over the 5 years between 1 January 2016, and 31 December 2020, a total of 431 patients were diagnosed with AL at the Hematology Centre of Armenia. Among this cohort, a substantial proportion, comprising 310 (71.9%) patients died. Our comprehensive analysis focused on a subset of 244 available patients with a median age of 59 years (age range 18–85 years) at the time of diagnosis and 112 (46%) of the patients were female. Among the diagnosed cases, 158 (65%) had AML, 56 (23%) ALL and 2 (1%) mixed phenotypes, while in 28 (11%) cases, the AL type remained unspecified (Table 1). A notable proportion accounting for 24% (58), passed away before frontline chemotherapy. Among these patients, 45% (26) refused treatment, while others experienced various complications due to disease progression. Among the patients who received chemotherapy, 34% (82) achieved CR. However, 29% (24) of these patients died during the first remission. The causes of death in these cases varied (acute heart failure, COVID-19, septic shock, pulmonary aspergillosis and acute respiratory failure). Notably, 20% (50) died during induction therapy. Of these cases, 29 (58%) were attributed to chemotherapy. The remaining patients passed away due to various reasons, including septic shock, acute heart failure, brain hemorrhage, acute respiratory failure, mesenteric venous thrombosis and multiple organ failure. Furthermore, 30 patients demonstrated resistance to the first-line therapy chose to discontinue further treatment and refused second-line therapy. In 10% (24), it was not possible to determine whether these patients achieved remission and what was the exact cause of death, underscoring the need for comprehensive documentation and standardised protocols for data collection (Figure 1). Among the patients who experienced relapse, only 7 (12%) patients reached second remission, indicating the challenges faced in managing recurrent disease. The 5-year OS was evaluated at 22% (Figure 2).

## Discussion

### Challenges of management of AML in Armenia

The incidence of AML in Armenia was evaluated at 1.12 per 100,000 which is notably lower compared with European developed countries and the United States (3–20 per 100,000) [11–14]. Among developing countries, Serbia has a higher incidence compared with Armenia (2.73 versus 1.12 per 100,000), and another developing country, Croatia, has a similar incidence with Armenia [14]. In our cohort, the incidence was 1.9 per 100,000. The prevalence of AML in Armenia was estimated at 7.4 per 100,000 populations as of January 1st, 2021, which reflects a relatively low but stable disease burden. This rate is lower than those reported in many European and North American populations, where prevalence often ranges from 12 to 20 per 100,000 [1, 14]. The observed difference may be attributed to factors such as younger population structure, underdiagnoses in older adults or variations in registry coverage and reporting practices.

Traditionally, the diagnosis of AML in Armenia relied on bone marrow cytochemistry. However, since 2010, the introduction of flow cytometry and the gradual adoption of immunophenotyping have made the latter the primary diagnostic method for AML, despite not being covered by state funding. In our cohort, out of 158 AML cases, 135 were diagnosed using immunophenotyping. Presently, cytogenetics and molecular genetics play pivotal roles in AML diagnostics, classification, risk stratification, prognosis and treatment protocol selection. While cytogenetic methods are available in Armenia, in our cohort they have been conducted in only 37% of patients due to high costs. Unfortunately, the availability of molecular genetic methods in Armenia is limited, making it impossible to detect gene mutations such as FLT3 and IDH, which play an important role in determining treatment strategies. FLT3 and IDH mutations occur in approximately 30% and 10%–15% of AML cases respectively [15, 16].

Several studies have demonstrated that FLT3- and IDH-targeted treatment modalities such as midostaurin, gilteritinib, quizartinib, enasidenib and ivosidenib prolong median OS [17–20]. Another essential option for AML management is core binding factor (CBF)-targeted therapy with GO. This targeted therapy has shown the potential to significantly increase long-term survival rates from less than 50% to 75% or more, making it a compelling option for all CBF AML patients [3]. The risk of CBF AML relapse can be reduced with additional allo-SCT, which is also beneficial for high-risk AML patients, those with relapsed/refractory disease and younger patients with intermediate/poor risk profiles [6].

In Armenia, the standard induction therapy for AML continues to be the time-tested ‘7 + 3’ regimen, consisting of 7 days of cytarabine and 3 days of anthracycline. This regimen, which has been a mainstay since the 1970s, is still widely used in many countries, including Armenia. Consolidation therapy primarily involves either 3–4 courses of high-dose cytarabine (HiDAC) or ‘7 + 3’ followed by maintenance therapy with the ‘5 + 5’ regimen (5 days of cytarabine + 6-mercaptopurine) every 6 weeks for 2 years.

The choice of treatment strategy often depends on the patient's socio-economic status, given that chemotherapy is only partially covered by the state. In our cohort, 32% received induction therapy with ‘7 + 3,’ and of those who achieved CR, 29% underwent consolidation therapy with HiDAC, while 71% followed the ‘7 + 3’ regimen, coupled with maintenance therapy. The relapse rates were 75% and 77%, respectively. A small fraction, 6% of patients, received treatment with low-dose cytarabine (LDAC) and 21% underwent other treatment regimens (Table 2). Unfortunately, the abovementioned targeted treatment modalities are not currently available in Armenia, and none of the patients in our cohort received targeted therapy.

**Figure 3.**
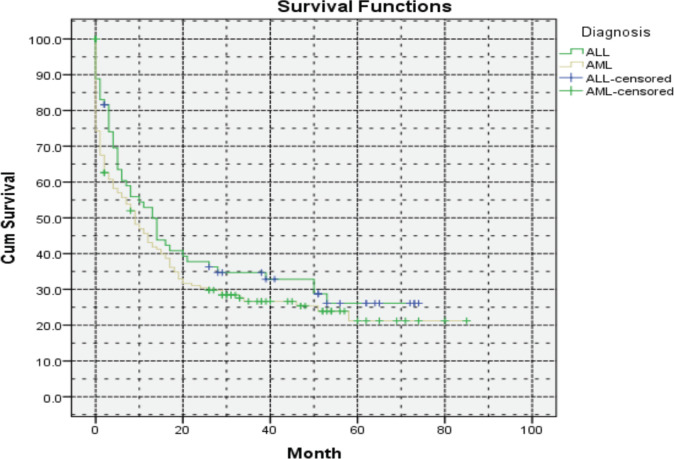
Five-year OS of ALL and AML patients in Armenia.

In summary, the integration of targeted therapies with induction/consolidation chemotherapy holds promise for improving outcomes in AML patients, particularly those with specific genetic mutations and the absence of these diagnostic and treatment modalities in Armenia has led to a decrease in OS compared to the developed world, with a 5-year OS rate of 21% (Figure 3).

### Challenges of management of ALL in Armenia

The incidence of ALL in Armenia was evaluated at 0.6 per 100,000, which is notably lower than in other countries, where it ranges from 2 to 3.5 per 100,000, including Australia, China, Costa Rica, Germany, Italy, Japan and the US [13, 21]. In our cohort, the incidence was 0.68 per 100,000, and the prevalence reached 10.8 per 100,000 as of 1^st^ January 2021. These findings highlight a relatively low rate of newly diagnosed cases, yet a higher cumulative burden possibly reflecting improved survival and treatment outcomes over time. Similar to AML patients, those with ALL face challenges in diagnostics and risk stratification due to limitations in cytogenetic and molecular genetic methods. In our cohort of 56 ALL patients, 98% received their diagnosis through immunophenotyping. The evaluation of Philadelphia chromosome positivity using the FISH method was conducted in 59% of patients. The mainstay treatment protocols during this period were GM ALL-2003 and GM ALL-1989. In our cohort, 23% of patients received GM ALL-2003, with 85% achieving CR. Additionally, 21% were treated with GM ALL-1989 13% with BFM-2000 or BFM-2007, 13% with CALGB 8811 and 11% with other regimens, with CR rates of 85%, 58%, 86%, 86% and 50%, respectively. Unfortunately, 59% of patients experienced relapse and only 50% of them received relapse therapy, with none achieving a second remission (Table 3).

The primary challenges in ALL treatment protocols lie in the high cost of drugs that are not covered by the state. Specifically, essential components of ALL treatment protocols, such as peg-asparaginase, rituximab and nelarabine are unaffordable for many patients, leading to some receiving treatment without these critical components. In the case of Ph-positive ALL patients, tyrosine kinase inhibitors are provided through the Max Foundation.

In the context of ALL, several new therapies, including bispecific T-cell engager blinatumomab, anti-CD22 antibody inotuzumab ozogomycin and CAR-therapy Tisagenlecleucel have been introduced and got FDA approvement. These treatment modalities have shown significant improvement in median OS and high remission rate [8–11, 22, 23].

Unfortunately, the unavailability of these crucial medications presents extreme challenges in the management of ALL, particularly in cases of early relapses and refractory disease. The 5-year OS in Armenia was evaluated at 26%, which is lower compared to the developed world (Figure 3).

Based on our analysis of the cohort, the challenges in AL management in Armenia can be categorised into two primary aspects:

Equipment: The limited availability of cytogenetic and molecular genetic methods poses significant challenges in achieving an accurate diagnosis, risk stratification and the utilisation of FDA-approved targeted therapies for ALs.

Treatment access: The absence of critical treatment options, including allo-SCT, GO, CPX-351, midostaurin, gilteritinib, quizartinib, enasidenib, ivosidenib, blinatumomab, inotuzumab ozogomycin, nelarabine and CAR-T therapy, as well as limited availability of essential chemotherapy drugs, deviates from standard treatment regimens, ultimately impacting the outcomes of AL. Here, it should be mentioned that within our cohort, 24% (58) of patients died of the disease before starting chemotherapy. Among them, 45% (26) declined treatment, primarily due to financial constraints. In some cases, patients were unaware of their diagnosis, and decisions were made by their relatives. Others faced various complications resulting from disease progression. Among the 82 patients who achieved CR, 29% (24) died during the first remission due to various treatment-related complications. Limited access to appropriate s care also played a role in these challenges. Access to supportive care also plays an important role in outcomes, such as the use of granulocyte colony-stimulating factors has shown to be highly effective in shortening the duration of chemotherapy-induced neutropenia, which is associated with a 20%–25% reduction in the incidence of febrile neutropenia. This reduction in febrile episodes translates to fewer hospitalisations and a decrease in the need for broad-spectrum antibiotics, further improving patient outcomes [24]. Antibiotics and antifungal treatments, such as posaconazole, are not consistently accessible or cost-effective for the country.

In this study, comprehensive data were collected from all cases of AL diagnosed at our centre, the only diagnostic institution for hematologic malignancies in Armenia. However, we should consider the potential for selection bias: the dataset does not account for possible cases of AL that remain undiagnosed, particularly in less accessible regions of the country. The absence of these cases from our analysis represents a notable limitation, as barriers like socio-economic constraints and limited disease awareness could influence the diagnosis and subsequent inclusion in our research. Consequently, while the study describes the situation for a part of the population, it simultaneously shows a selection bias. This underscores a need for expanded healthcare outreach and improved awareness of the disease throughout Armenia. Our findings, therefore, provide an essential, albeit incomplete, perspective on the mortality of AL in Armenia.

In conclusion, the limited availability of modern diagnostic and treatment modalities in Armenia has resulted in inferior outcomes of AL with a lower median OS compared with the developed world. Addressing these challenges requires efforts to enhance diagnostic and treatment accessibility giving all patients an equal chance of overcoming this malignant disease regardless of their socio-economic background, and trying to achieve outcomes comparable to those in the developed world.

## List of abbreviations

AL, acute leukaemia; ALL, acute lymphoblastic leukaemia; allo-SCT, allogeneic stem cell transplantation; AML, acute myeloid leukaemia; CNS, central nervous system; OS, overall survival; PLT, platelets; WBC, white blood cells.

## Funding statement

This research did not receive any specific grant from funding agencies in the public, commercial or not-for-profit sectors.

## Conflicts of interest

The authors have stated that they have no conflicts of interest.

## Figures and Tables

**Table 1.** Demographic and diagnostic profile of patients with AL.

Characteristics	AL total	AML	ALL	AL, unspecified	AL, mixed phenotype
Number of patients	244	158	56	28	2
Gender (female %)	46%	41%	46%	63%	50%
Median age (range)	58	56	50	69	45.5
Median age range	18–86	20–84	18–84	49–86	35–56
WBC median count	36 × 10^9^/L	53 × 10^9^/L	45× 10^9^/L	44 × 10^9^/L	6.58 × 10^9^/L
WBC range	0.17–585	0.17–358	0.79–378.12	1.16–585	6.57–6.6
PLT median count	65 × 10^9^/L	69 × 10^9^/L	118 × 10^9^/L	61 × 10^9^/L	10 × 10^9^/L
PLT range	3–393	3–393	4–331	6–301	7–13
Splenomegaly (%)	44	37	63	44	67
Primary CNS involvement (%)	3	1	11	0	0
Lymphadenopathy (%pts)	52%	51%	68%	30%	67%
Immunophenotyping performed in (%pts)	80%	85%	98%	11%	100%
Cytogenetics performed in (%pts)	39%	37%	59%	7%	33%
Cytochemistry performed in (%pts)	N/A	15%	2%	N/A	0

AL; acute leukaemia, AML; acute myeloid leukaemia, ALL; acute lymphoblastic leukaemia, WBC; white blood cell, PLT; platelet, CNS; central nervous system

**Table 2.** Management and outcomes of AML.

Treatment protocols	*N* (%)	CR *N* (%)	Relapse *N* (%)	Relapse therapy *N* (%)	Second CR *N* (%)
Induction therapy with “7 + 3”	77 (32%)	42 (55%)	32 (76%)	20 (65%)	2 (10%)
Consolidation therapy with HiDAC (after reaching CR with “7 + 3”)		12 (29%)	9 (75%)	7 (78%)	2 (29%)
Consolidation therapy with “7 + 3” followed with maintenance therapy with “5 + 5” (after reaching CR with “7 + 3”)		30 (71%)	23 (77%)	13 (59%)	3 (23%)
LDAC	15 (6%)	5 (33%)	4 (80%)	N/A	0
Other	16 (21%)	N/A	N/A	N/A	N/A

**Table 3.** Management and outcomes of ALL.

Treatment protocols	*N* (%)	CR *N* (%)	Relapse rate Pts number N (%)	Relapse therapy *N* (%)	Second CR *N* (%)
GM ALL 2003	13 (23%)	11 (85%)	8 (73%)	5 (71%)	0
GM ALL 1989	12 (21%)	7 (58%)	5 (71%)	3 (60%)	0
BFM 2000 or 2007	7 (13%)	6 (86%)	4 (67%)	2 (67%)	0
CALGB 8811	7 (13%)	6 (86%)	3 (50%)	0	0
Other	6 (11%)	3 (50%)	n/a	n/a	n/a
